# Reciprocal circulation pattern of SARS-CoV-2 and influenza viruses during the influenza seasons 2019/2020 and 2020/2021 in the Bavarian Influenza Sentinel (Germany)

**DOI:** 10.1017/S0950268821002296

**Published:** 2021-10-26

**Authors:** Susanne Heinzinger, Ute Eberle, Hildegard Angermeier, Jennifer Flechsler, Regina Konrad, Alexandra Dangel, Carola Berger, Annika Sprenger, Sabrina Hepner, Barbara Biere, Bernhard Liebl, Nikolaus Ackermann, Andreas Sing

**Affiliations:** 1 Department of Public Health Microbiology, Bavarian Health and Food Safety Authority, Oberschleißheim, Germany; 2 Department of Virology, Bavarian Health and Food Safety Authority, Oberschleißheim, Germany; 3 State Institute of Public Health, Bavarian Health and Food Safety Authority, Oberschleißheim, Germany; 4 National Reference Center for Influenza, Robert Koch Institute, Berlin, Germany; 5 Ludwig Maximilians-Universität, Munich, Germany

**Keywords:** Influenza, COVID-19, SARS-CoV-2, RT-PCR, surveillance

## Abstract

The corona virus disease-2019 (COVID-19) pandemic began in Wuhan, China, and quickly spread around the world. The pandemic overlapped with two consecutive influenza seasons (2019/2020 and 2020/2021). This provided the opportunity to study community circulation of influenza viruses and severe acute respiratory syndrome-coronavirus-2 (SARS-CoV-2) in outpatients with acute respiratory infections during these two seasons within the Bavarian Influenza Sentinel (BIS) in Bavaria, Germany. From September to March, oropharyngeal swabs collected at BIS were analysed for influenza viruses and SARS-CoV-2 by real-time polymerase chain reaction. In BIS 2019/2020, 1376 swabs were tested for influenza viruses. The average positive rate was 37.6%, with a maximum of over 60% (in January). The predominant influenza viruses were Influenza A(H1N1)pdm09 (*n* = 202), Influenza A(H3N2) (*n* = 144) and Influenza B Victoria lineage (*n* = 129). In all, 610 of these BIS swabs contained sufficient material to retrospectively test for SARS-CoV-2. SARS-CoV-2 RNA was not detectable in any of these swabs. In BIS 2020/2021, 470 swabs were tested for influenza viruses and 457 for SARS-CoV-2. Only three swabs (0.6%) were positive for Influenza, while SARS-CoV-2 was found in 30 swabs (6.6%). We showed that no circulation of SARS-CoV-2 was detectable in BIS during the 2019/2020 influenza season, while virtually no influenza viruses were found in BIS 2020/2021 during the COVID-19 pandemic.

## Introduction

On 11 March 2020, the WHO declared the coronavirus disease-2019 (COVID-19) outbreak a pandemic. The pandemic started in Wuhan/China rapidly spreading all over the world [1–3]. The first cases in the WHO European Region occurred in France on 24 January 2020 with the onset of symptoms in the first patient on 17 January [4]. In Germany, the first case was announced to the Bavarian Health and Food Safety Authority (LGL), Bavaria in a calendar week (CW) 5 (27 January 2020) after a job-related contact to a Chinese index patient within a company in the greater area of Munich [5–7].

The Bavarian Influenza Sentinel (BIS) was established in 2009 in Bavaria, Germany by the LGL to complement syndromic surveillance with virological data [8]. The objectives of BIS are to determine the onset and duration of the influenza season and to identify the circulating influenza viruses and their subtypes, the age groups (AGs) affected and the proportion of influenza-like illness (ILI) in outpatients with acute respiratory infections (ARI).

Moreover, in this study, we investigated the concurrent circulation of influenza viruses and SARS-CoV-2 in two consecutive influenza seasons (2019/2020 and 2020/2021) to study the impact of the COVID-19 pandemic and its interventions on the spread of influenza viruses and SARS-CoV-2.

## Methods

### BIS structure

About 75 general practitioners spread throughout Bavaria voluntarily participate in BIS. Every week, from about CW 38 to about CW 14 (end of September until the end of March) of each influenza season they send one sample (nasal or throat swab) each from two randomly selected ARI patients to our laboratory. Swabs and associated medium were obtained from either Sigma Virocult (Medical Wire & Equipment Co. (Bath) Ltd, UK) or Yocon (Yocon Biology Technology Company, Beijing, China) and stored at 4 °C until further laboratory analysis. In addition, information about the disease was collected via a standardised questionnaire. Age, gender, clinical symptoms (time of onset, fever about 38 °C, cough, pneumonia and other symptoms) were reported.

### Syndromic case definitions

ARI is defined as the acute onset of symptoms (cough and/or sore throat and/or shortness of breath and/or runny nose with or without fever) in a patient, and clinical assessment of an underlying infection; ILI according to the WHO as an acute respiratory illness with a fever above 38° C, cough and acute onset of illness [9, 10].

### Laboratory diagnostics

Detection of influenza RNA was conducted shortly after sample collection. In season 2019/2020 a QIAamp Bio Robot kit (QIAGEN, Hilden, Germany) was used for RNA extraction on a Hamilton Microlab Star (Hamilton, Bonaduz, Switzerland) followed by RealStar^®^ Influenza Screen & Type RT-PCR Kit 4.0 on a Bio-Rad CFX96 Touch Real-Time PCR Detection System (Bio-Rad, Feldkirchen, Germany). Specimens were further tested with real-time polymerase chain reaction (RT-PCR) to identify the influenza virus (Influenza A, subtypes (H1N1)pdm09 and H3N2 and Influenza B, lineage Victoria and Yamagata) [11, 12]. SARS-CoV-2 diagnosis was performed as previously described [13]. In season 2020/2021 AltoStar^®^ Automation System AM16 was used for RNA extraction followed by RealStar® Influenza Screen & Type RT-PCR Kit 4.0 and RealStar^®^ SARS-CoV-2 RT-PCR Kit on a Bio-Rad CFX96 Real-Time PCR Detection System (Bio-Rad, Feldkirchen, Germany). Different methods for RNA extraction and for the analysis of influenza viruses and SARS-CoV-2 were validated internally and externally in their equivalent quality by interlaboratory tests. Due to time-critical SARS-CoV-2 diagnostics Xpert^®^ Xpress SARS-CoV-2, Cepheid, USA was also in use as a rapid test. Afterwards, samples were stored at −20 °C.

### Data analysis

Real-time PCR data were evaluated with Bio-Read CFX-Maestro software. For this, the threshold was manually set within the exponential phase of the detection curve. Further data analysis was performed using Spyder 4.1.4. (Python 3.8). The positive predictive value (PPV) for a given symptom was calculated as the number of patients with that symptom divided by the number of all patients.

### Ethics approval

Ethics approval was obtained (Ethik-Kommission Nr. 15053). The authors assert that all procedures contributing to this work comply with the ethical standards of the relevant national and institutional committees on human experimentation and with the Helsinki Declaration of 1975, as revised in 2008.

### LGL mass testing

The SARS-CoV-2 analyses performed by the LGL included outbreak investigations and contact person tests initiated by the 76 local public health authorities in Bavaria.

## Results

### General considerations

While in 2019/2020 58 doctors from 42 out of 76 counties and from all seven government districts in Bavaria participated in BIS, in 2020/2021 only 41 (70.7%) from 31 counties and seven government districts were involved in BIS (Table 1). In 2019/2020 they collected 1376 samples; in 2020/2021 only 470 (34.2%) samples were obtained. At least one sample per week was sent by 42 general practitioners in 2019/2020, summing up to 1245 samples (90.5% of all samples). In 2020/2021, 12 general practitioners sent at least one sample per week to the LGL, summing up to 329 samples (70.0% of all samples).

**Table 1.** Basic data of the BIS 2019/2020 and 2020/2021 in Bavaria, Germany

	BIS 2019/2020	BIS 2020/2021	Interseasonal change
Number of physicians	58	41	70.7%
With at least 1 sample per week	42	12	
Proportion of physicians with 1 sample per week to all physicians	72.4%	29.3%	
Number of samples			
For influenza virus analysis	1376	470	34.2%
From physicians with at least 1 sample per week	1245	329	
Proportion of influenza samples from physicians with 1 sample per week to all influenza samples	90.5%	70.0%	
For SARS-CoV-2 analysis	610	457	
Proportion of SARS-CoV-2 analysis to the influenza virus analysis	44.3%	97.2%	

BIS, Bavarian Influenza Sentinel.

### Influenza surveillance

**Fig. 1.**
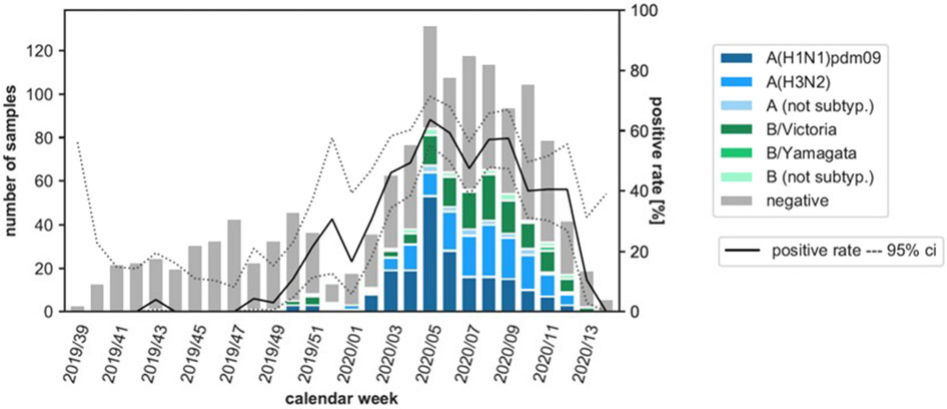
Number of samples analysed for influenza viruses, detected subtypes of influenza viruses and proportion of positive influenza virus detections (positivity rate) by CW in the Bavarian Influenza Sentinel 2019/2020 in Bavaria, Germany. Dark blue bar: Influenza A(H1N1)pdm09, blue bar: Influenza A(H3N2), light blue bar: Influenza A not further subtyped, dark green bar: Influenza B Victoria lineage, green bar: Influenza B Yamagata lineage, light green bar: Influenza B not further subtyped, grey bar: negative swabs. Black line: positive rate, dashed lines: upper and lower 95% confidence interval (ci). Note: The first lockdown in Germany started in CW 12/2020.

In the flu season 2019/2020 (CW 39/2019 – CW 14/2020: 28 weeks), 1376 specimens were tested for influenza viruses in total. 517 (37.6%) of these samples were positive: 202 (39.1%) Influenza A(H1N1)pdm09 viruses, 144 (27.9%) Influenza A(H3N2), 18 (3.5%) Influenza A not further subtyped, 129 (25.0%) Influenza B Victoria lineage, 22 (4.3%) Influenza B Yamagata lineage and 2 (0.4%) Influenza B not further subtyped. The positive rate began to increase from CW 48 (Fig. 1). The peak of this epidemic was in CW 5 with a positive rate of more than 60%. According to the definition of the Robert Koch-Institute (RKI), the start of the seasonal influenza epidemic is set for CW 51/2019, the end for CW 12/2020 [14].

In the influenza season 2020/2021 (CW 38/2019 – CW 11/2021: 27 weeks), 470 specimens were tested for influenza viruses. Most interestingly, only three (0.6%) samples were positive: one Influenza B Victoria lineage in CW 52/2020 and two Influenza A(H3N2) in CW 02 and CW 10/2021.

### SARS-CoV-2 Surveillance

In the BIS 2019/2020, 610 swabs (44.3% of all samples) for which sufficient material was available were retrospectively tested for SARS-CoV-2 (Table 1). The number of SARS-CoV-2 samples tested weekly largely paralleled the number of samples tested weekly for influenza. A median of 47% (2–65%) of influenza samples was mapped. No positive results were obtained.

**Fig. 2.**
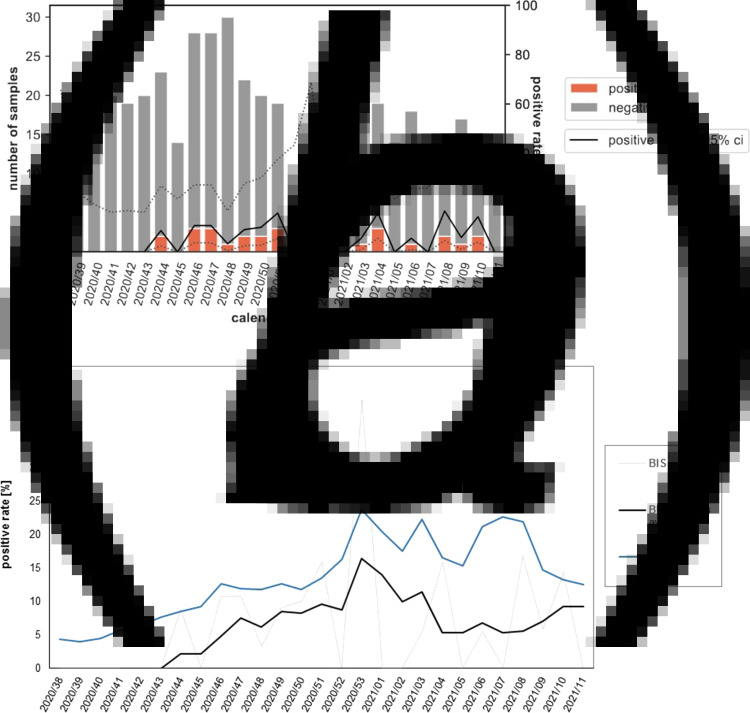
(a)Number of samples analysed for SARS-CoV-2 viruses and proportion of positive SARS-CoV-2 detections (positive rate) by CW in the Bavarian Influenza Sentinel 2020/2021 in Bavaria, Germany. Grey bar: negative swabs, orange bars: SARS-CoV-2 positive swabs. Black line: positive rate, dashed lines: upper and lower 95% confidence interval (ci). Note: The second lockdown-‘light’ in Germany started in CW 44/2020. It was replaced by a hard lockdown in CW 51/2020, which lasted until CW 10/2021. (b) SARS-CoV-2 positive rate by CW in the Bavarian Influenza Sentinel 2020/2021 (BIS, dotted line), the four-week average positive rate in BIS (black line) and the positive rate from the Bavarian Health and Food Safety Authority (LGL) mass testing (blue line).

During the 2020/2021 influenza season, 457 samples (97.2% of all samples) were analysed for SARS-CoV-2 (Table 1). Of these, 30 samples (6.6%) were tested positive, spread over the entire period (Fig. 2a). The four-week average positive rate in BIS largely paralleled the positive rates of LGL mass testing (Fig. 2b), especially in CW 47/2020 to 03/2021, where the positive rate in BIS was only 29.3–48.7% lower than that of mass testing (244 519 respiratory samples).

### Analysis by age and gender

**Fig. 3.**
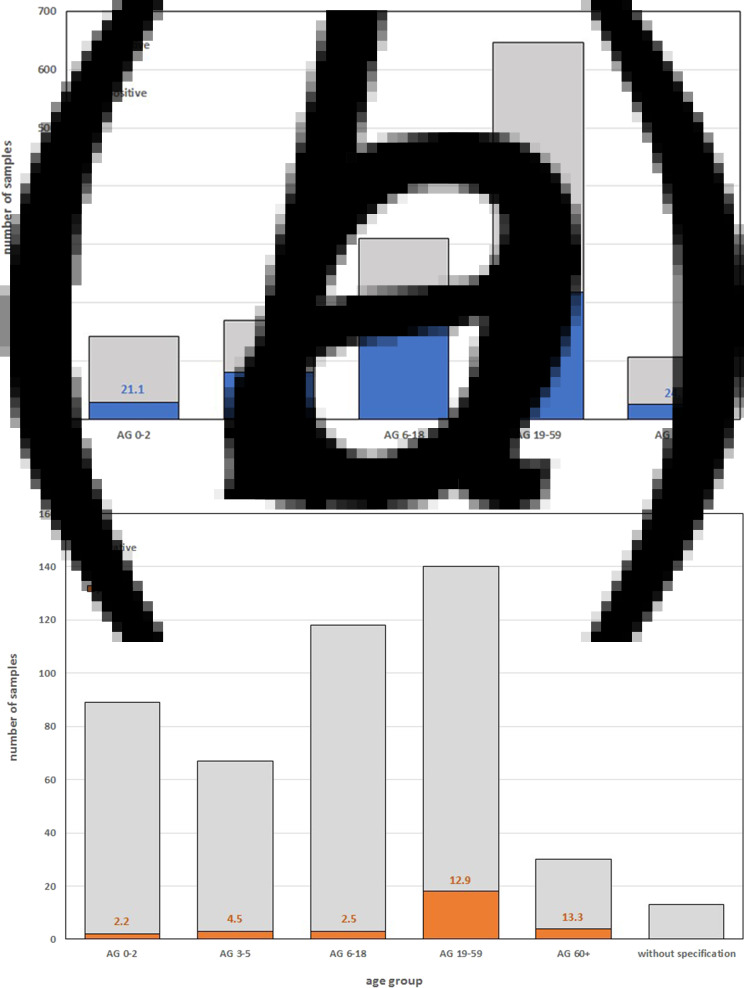
Number of swabs with positive and negative (a) influenza virus detection in Bavarian Influenza Sentinel (BIS) 2019/2020 and (b) SARS-CoV-2 detection in BIS 2020/2021 in different AGs. The coloured numbers in the bars indicate the respective positive rates.

Most samples were collected in the 19–59 AG in both flu seasons (Fig. 3a). In BIS 2020/2021 considerably more swabs from children (AG 0–18) were sent to the LGL (Fig. 3b).

In BIS 2019/2020 the highest influenza-positive rate was found in AG 6–18 (52.1%), followed by AG 3–5 (47.6%) (Fig. 3a). In BIS 2020/2021 only three (0.6%) samples were positive for influenza virus (two in AG 0–2 and one in AG 6–18) (data not shown). The highest positive rate of SARS-CoV-2 in BIS 2020/2021 was found in the AG 60+ (13.3%), followed by AG 19–59 (12.9%) and 2.2–4.5% in AG 0–18 (Fig. 3b).

A gender-dependent effect on influenza virus or SARS-CoV-2 infection was not observed in these studies.

### Analysis by symptoms

In BIS 2019/2020 influenza virus infection was associated with cough (95% of influenza-positive individuals), fever (91%), and acute onset (92%). Of all 1376 ARI patients, 829 (60.2%) met the ILI case definition, including 423 with influenza infection (82% of influenza-positive ARI patients) and 406 without influenza infection (47% of influenza-negative ARI patients) (Table 2). Influenza is often accompanied by other symptoms (72%), including, for example, headache, which was reported by 45% of outpatients. These other symptoms are not very specific for influenza. They are also reported by 73% of ARI patients without influenza.

**Table 2.** Symptoms of patients infected with influenza virus or SARS-CoV-2 in the BIS 2019/2020, respectively 2020/2021 in Bavaria, Germany and PPV of each symptom

Symptoms	Influenzavirus BIS 2019/2020					SARS-CoV-2 BIS 2020/2021				
	Positive		Negative		PPV	Positive		Negative		PPV
	number (*N* = 517)	% of total	number (*N* = 859)	% of total		number (*N* = 30)	% of total	number (*N* = 427)	% of total	
Cough	491	95%	672	78%	42%	20	67%	240	63%	8%
Acute onset	474	92%	707	82%	40%	21	70%	349	91%	6%
Fever	469	91%	582	68%	45%	17	57%	227	59%	7%
ILI	423	82%	406	47%	51%	10	33%	109	28%	8%
Other symptoms:	373	72%	624	73%	37%	16	53%	265	69%	6%
Muscle pain	89	17%	154	18%	37%	1	3%	18	5%	5%
Pain in the limbs	107	21%	189	22%	36%	5	17%	46	12%	10%
Sore throat	133	26%	297	35%	31%	8	27%	100	26%	7%
Headache	234	45%	384	45%	38%	4	13%	97	25%	4%
Pneumonia	15	3%	33	4%	31%	1	3%	5	1%	17%

BIS, Bavarian Influenza Sentinel; PPV, positive predictive value.

In contrast, in BIS 2020/2021 ARI patients with SARS-CoV-2 were less likely to show typical ILI symptoms (only 33%). There was also little difference in the frequency of cough, fever or acute onset compared to SARS-CoV-2-negative ARI patients. In our calculations, PPV indicates the likelihood that a patient with a given symptom, was infected with a virus. For example, in BIS 2019/2020, 42% of ARI patients with cough were truly infected with influenza viruses, whereas in BIS 2020/2021, the PPV that patients with a cough had SARS-CoV-2 was only 8%.

## Discussion

The abrupt end of the influenza season in BIS 2019/2020 was also observed on a national level by the Robert Koch Institute [15] and as well in other European countries [16, 17], most likely due to the Public Health response to the SARS-CoV-2 pandemic (first lockdown in Germany in March 2020).

While during normal influenza seasons most influenza cases occur in the northern hemisphere in late January and February, a very unusually low influenza virus activity was observed worldwide during the 2020/2021 season (background activity) [18–21]. Typically, influenza seasons are characterised by several factors including the prevailing influenza virus strains, background immunity and vaccine effects; the 2020/2021 season was additionally influenced by the public health response to the SARS-CoV-2 pandemic, e.g. mandatory masks, social distancing or hand hygiene. From CW 40/2020 to CW 11/2021, no influenza cases were detected in the 3288 samples sent to RKI influenza surveillance. Moreover, in the reporting data according to the German Infection Protection Act, only 479 laboratory-confirmed influenza cases have been reported to the RKI since week 40/2020. In the same period of the 2019/2020 flu season, 165 036 cases were notified [21]. ECDC also noted in Flu News, that for the 2020/2021 season 37 of 29 468 (0.1%) sentinel samples and 704 of 528 209 (0.1%) non-sentinel samples were positive for influenza virus [20]. A decrease in influenza cases was also noticeable in BIS (0.7% of the samples in 2020/2021). Even taking into account the lower number of submissions because of a decrease of participating physicians and of total sample numbers, an extrapolation would suggest only nine influenza cases per 470 smears, which would correspond to an average positive rate of 2% in BIS 2020/2021, compared to 38% in 2019/2020.

Possible reasons for lowered submissions were probably an initial work overload of physicians due to the COVID-19 pandemic allowing less time for participation in BIS. Later in the season, additional factors might have become also important, e.g. the lower incidence of ARI due to the hygiene measures in place and a decrease in ARI patients at private practitioners, as patients may have turned directly to a test centre when they had ARI symptoms and no longer consulted their general practitioner.

Our retrospective analysis for SARS-CoV-2 around the first occurrence of the first COVID-19 case in Germany [1] as well as studies from China and the USA failed to detect positive SARS-CoV-2 patients from before the first verified COVID-19 case in the respective country or over a period shortly thereafter [13, 22–26]. One reason could be that the incidence of COVID-19 was too low to be detected by syndromic surveillance systems.

In the 2020/2021 influenza season, 7% of oropharyngeal swabs testing positive for SARS-CoV-2 in BIS were detected throughout the season. The RKI also detected SARS-CoV-2 in 247 (8%) of the 3281 sentinel samples examined [21]. In both sentinels, it remains to be noted that the medical practices were not completely representative of the Bavarian or German population and that the SARS-CoV-2 circulation was also not evenly distributed across the medical practices or regions. The elderly were particularly affected by SARS-CoV-2, as reflected in the increased positive rate in BIS 2020/2021 (13.3% in AG 60+, 12.9% in AG 19–59 *vs.* 2.9% in AG 0–18) and the greatly increased 7-day incidence of COVID-19 cases in Germany in the 80+ AG, shown by the RKI, followed by that of AG 15–64 [27]. Furthermore, the ECDC COVID-19 surveillance report for Europe showed that as of 1 August 2020, most COVID-19 cases were found in AG 20–59, with most hospitalised people in the 70+ AG [28]. Symptomatically, the two viral infections differed in that ARI patients with influenza fulfilled the ILI definition (simultaneous presence of acute onset, cough and fever) in 82% of cases, whereas SARS-CoV-2 patients had ILI in only 33% of cases. This means that the simultaneous presence of cough, fever and acute onset did not reliably indicate SARS-CoV-2 infection.

In addition to the BIS, the incidence of SARS-CoV-2 was also monitored in Bavaria by mass testing performed by the LGL laboratory on behalf of the 76 Bavarian local public health authorities. The nearly parallel development of the positivity rate in the season 2020/2021 in both systems regardless of the different objectives and composition of the tested populations (e.g. symptomatic ARI patients *vs.* mainly outbreak and contact person testing) underlines the robustness of the BIS data at least for SARS-CoV-2.

Among the limitations outlined above are the lower number of physicians and samples in the 2020/2021 season, which may have underestimated the true incidence of influenza infections, and the retrospective analysis of SARS-CoV-2 in BIS 2019/2020, which may have underestimated the true incidence of SARS-CoV-2.

The abrupt end of the 2019/2020 influenza season and the near absence of influenza virus circulation in the 2020/2021 season led us to suggest that the public health response to the coronavirus pandemic may have worked even better against influenza than against SARS-CoV-2, as SARS-CoV-2 was detected at significant levels in ARI patients in the 2020/2021 BIS season.

In summary, we showed that no circulation of SARS-CoV-2 was detectable in BIS during the 2019/2020 influenza season, while virtually no influenza viruses were found in BIS 2020/2021 during the COVID-19 pandemic.

## Data Availability

For data availability, readers can contact the authors if they want access to such materials. Data are only available in anonymised form.
