# Reciprocal circulation pattern of SARS-CoV-2 and influenza viruses during the influenza seasons 2019/2020 and 2020/2021 in the Bavarian Influenza Sentinel (Germany) – CORRIGENDUM

**DOI:** 10.1017/S0950268821002466

**Published:** 2022-02-11

**Authors:** Susanne Heinzinger, Ute Eberle, Hildegard Angermeier, Jennifer Flechsler, Regina Konrad, Alexandra Dangel, Carola Berger, Annika Sprenger, Sabrina Hepner, Barbara Biere, Bernhard Liebl, Nikolaus Ackermann, Andreas Sing

**Affiliations:** 1 Department of Public Health Microbiology, Bavarian Health and Food Safety Authority, Oberschleißheim, Germany; 2 Department of Virology, Bavarian Health and Food Safety Authority, Oberschleißheim, Germany; 3 National Reference Center for Influenza, Robert Koch Institute, Berlin, Germany State Institute of Public Health; 4 Bavarian Health and Food Safety Authority, Oberschleißheim, Germany; 5 Ludwig Maximilians-Universität, Munich, Germany

The original version of this article was published with two of the author affiliations listed in the incorrect order and subsequently three authors were listed with institutions incorrectly assigned to them.

The transposed institutions were ‘National Reference Center for Influenza, Robert Koch Institute, Berlin, Germany State Institute of Public Health’ and ‘Bavarian Health and Food Safety Authority, Oberschleißheim, Germany’.

The affected authors are listed with their correct affiliations below

Barbara Biere: National Reference Center for Influenza, Robert Koch Institute, Berlin, Germany

Bernhard Liebl: State Institute of Public Health, Bavarian Health and Food Safety Authority, Oberschleißheim, Germany; Ludwig Maximilians-Universität, Munich, Germany

Andreas Sing: Department of Public Health Microbiology, Bavarian Health and Food Safety Authority, Oberschleißheim, Germany; State Institute of Public Health, Bavarian Health and Food Safety Authority, Oberschleißheim, Germany; Ludwig Maximilians-Universität, Munich, Germany

The original version of the article has now been corrected.
